# Supplementary material for: Applications of Artificial Intelligence for Metastatic Gastrointestinal Cancer: A Systematic Literature Review
Source: Cancers (Basel). 2025 Feb 6;17(3):558. doi: 10.3390/cancers17030558 (PMC11817159; doi:10.3390/cancers17030558)
Supplement: Supplementary file 1 [file cancers-17-00558-s001.zip › S2.pdf]

Scopus and Google Scholar terms:
(("Gastrointestinal" OR "Digestive System" OR "Esophageal" OR "Stomach" OR "Colorectal" OR "Liver" OR "Biliary Tract" OR "Peritoneal" OR "Pancreatic") AND neoplasm) AND (metastasis OR "Neoplasm Metastasis") AND ("Artificial Intelligence" OR "Machine Learning" OR "Artificial Neural Networks" OR "Bayesian Learning" OR "Classification Algorithm" OR "Deep Learning" OR "Fuzzy System" OR "Hidden Markov Model" OR "Learning Algorithm" OR "Supervised Machine Learning" OR "Support Vector Machine" OR "Unsupervised Machine Learning"))

MeSH terms:
Gastrointestinal Neoplasms, Digestive System Neoplasms, Esophageal Neoplasms, Stomach Neoplasms, Colorectal Neoplasms, Liver Neoplasms, Rectal Neoplasms, Biliary Tract Neoplasms, Pancreatic Neoplasms, Peritoneal Neoplasms, Cancer, Metastasis, Neoplasm Metastasis, Artificial Intelligence, Machine Learning, Artificial Neural Network, Bayesian Learning, Classification Algorithm, Deep Learning, Fuzzy System, Hidden Markov Model, Learning Algorithm, Supervised Machine Learning, Support Vector Machine, Unsupervised Machine Learning.
